# QTL Analysis Revealed One Major Genetic Factor Inhibiting Lesion Elongation by Bacterial Blight (*Xanthomonas oryzae* pv. *oryzae*) from a *japonica* Cultivar Koshihikari in Rice

**DOI:** 10.3390/plants11070867

**Published:** 2022-03-24

**Authors:** Shameel Shah, Hiroaki Tsuneyoshi, Katsuyuki Ichitani, Satoru Taura

**Affiliations:** 1 Graduate School of Agriculture Science Forestry and Fisheries, Kagoshima University, Kagoshima 890-0065, Japan; shahshameel@hotmail.com (S.S.); ichitani@agri.kagoshima-u.ac.jp (K.I.); 2 Faculty of Agriculture, Kagoshima University, Kagoshima 890-0065, Japan; a-friend-1n-need@docomo.ne.jp; 3 The United Graduate School of Agriculture Sciences, Kagoshima University, Kagoshima 890-0065, Japan; 4 Division of Gene Research, Kagoshima University, Kagoshima 890-0065, Japan

**Keywords:** pathogen, DNA marker, composite interval mapping (CIM), simple interval mapping (SIM), quantitative trait loci, horizontal resistance

## Abstract

*Xanthomonas oryzae* pv. *oryzae* (*Xoo*) is a pathogen that has ravaged the rice industry as the causal agent of bacterial blight (BB) diseases in rice. Koshihikari (KO), an elite *japonica* cultivar, and ARC7013 (AR), an *indica* cultivar, are both susceptible to *Xoo*. Their phenotypic characteristics reveal that KO has shorter lesion length than that of AR. The F_2_ population from KO × AR results in continuous distribution of lesion length by inoculation of an *Xoo* race (T7147). Consequently, quantitative trait loci (QTL) mapping of the F_2_ population is conducted, covering 12 chromosomes with 107 simple sequence repeat (SSR) and insertion/deletion (InDel) genetic markers. Three QTLs are identified on chromosomes 2, 5, and 10. Of them, *qXAR5* has the strongest resistance variance effect of 20.5%, whereas *qXAR2* and *qXAR10* have minor QTL effects on resistance variance, with 3.9% and 2.3%, respectively, for a total resistance variance of 26.7%. The QTLs we examine for this study differ from the loci of BB resistance genes from earlier studies. Our results can help to facilitate understanding of genetic and morphological fundamentals for use in rice breeding programs that are more durable against evolving *Xoo* pathogens and uncertain climatic temperature.

## 1. Introduction

Access to a healthy diet is humanity’s most important need. The United Nations sustainable development goals aim to eliminate hunger by 2030. However, this world is plagued with hunger. Reaching this important goal is further complicated by climate change, population increase, and increases in pests and diseases in both humans and crops. To provide food security, a greater need exists for modern technology and techniques. Rice (*Oryza sativa* L.) is one of the most consumed foods in the world apart from wheat and maize, with 508.7 million tons produced during 2010–2021 [1]. As a staple food, rice feeds more than half of the human population [2,3]. Apart from being used as food, rice has great traditional and cultural importance in Asian countries. In Japan, rice is regarded as the essence of culture. It is cultivated from Hokkaido, the most northern prefecture of Japan, to the most southern regions of Japan in Okinawa. Like economically developed countries, economically developing island states such as Fiji also depend on rice not merely for food security but also to stimulate the economy through exportation.

Biotic stressors such as pathogens strongly affect rice production. *Xanthomonas oryzae* pv. *oryzae* (*Xoo*), a pathogen that has ravaged the rice industry as the causal agent of bacterial blight disease (BB) in rice and grass species, can cause up to 50% annual yield loss [4,5]. Initially, BB was reported in 1884 in southern Japan [6]. To combat this pathogen, an integrated pest management strategy is needed. Currently cultural, chemical, biological, and molecular control methods have been devised. Cultural control methods include proper nursery drainage, removal of diseased plants, weeds and debris, and burning rice straw left from the prior season [7]. Chemical control is a temporary measure that causes environmental pollution and adverse degradation [8,9]. Bactericides such as bismerthiazole and streptomycin have been used widely to control the *Xoo* pathogen in parts of China, which has exacerbated the problem of antimicrobial resistance development [10,11]. Toxic residues left by chemical control in the soil’s rhizosphere can engender development of antibiotic and chemical resistance in other microorganisms, which is expected to be devastating if it were to become zoonotic, causing serious problems in the public health sector. Biological approaches are regarded as ecologically friendly and good long-term solutions [12,13]. According to Ji et al., *Lysobacter antibioticus* has the potential to be used for biocontrol because it suppresses *Xoo* pathogen growth [14]. Plant growth promoting rhizobacteria such as *B. pumilus* SE34 and *B. subtilis* GBO3 has been shown to induce systematic resistance against *Xoo* pathogens while helping to enhance nutrient uptake and yields as shown in in vitro studies [15]. In contrast, the most effective control method for BB is genetic selection for resistant rice cultivars [16,17,18]. To date, more than 40 known resistance genes (*R* genes) have shown resistance to different strains of *Xoo* pathogen in rice cultivars, wild relatives, and some mutation populations [19].

Plant resistance is generally categorized as vertical or horizontal. According to Acquaah [20], vertical resistance is characterized by several features: it is based on a hypersensitivity reaction; it is race specific or pathotype specific in its effects; its inheritance is based on a major gene; and its effects in agricultural applications are usually not durable. Horizontal resistance is also known as partial resistance, field resistance, or race-non-specific resistance. This resistance is typically not complete. It is effective against all genotypes of the pathogen species. Most reported *R* genes against *Xoo* confer vertical resistance. *Xoo*, similar to any other pathogen, can evolve and persist in the environment until it finds a suitable host. This capability makes it difficult to create a durable resistance because major *R* genes eventually “breakdown” [8,19], creating doubts for future food insecurities. For instance, the *Xa4* gene has been used extensively in most elite rice cultivars, enabling it to provide defense against the *Xoo* pathogen. However, many cultivars that have been developed, which carry only the *Xa4* gene, have become susceptible to new strains of *Xoo*. They no longer provide resistance [21,22].

Moreover, climate change and increased temperatures can be expected to promote further complications that will make general *R* genes more susceptible to the *Xoo* pathogen [23,24]. Durability of BB *R* genes such as *Xa4* show decreased effectiveness in controlling BB disease at higher temperatures [25]. This important shortcoming has spurred further exploration of horizontal resistance. This form of resistance is associated with polygenic genes controlled by multiple loci [26]. Ramalingam et al. and Li et al. reported that quantitative resistance, when compared to qualitative resistance, is regarded as providing higher durability against evolving and stronger virulence pathogens [27,28].

A Japanese rice cultivar, Koshihikari (KO), is known to be susceptible to all Japanese *Xoo* races [29]. However, our field observations over many years have revealed that, although permitting invasion of *Xoo* races into leaves, KO inhibits the progression of lesion growth against different *Xoo* races. This characteristic of KO presents a clear contrast to very long lesion length by *Xoo* of an Indian cultivar ARC7013 (AR), which also lacks *R* genes. So, why does this phenomenon occur when both cultivars are susceptible? This research seeks to ascertain if this phenomenon is caused by horizontal resistance genes that are responsible for providing Koshihikari this innate protection against BB disease. Moreover, because both KO and AR lack major vertical resistance genes, they would be good genetic materials for analyzing horizontal resistance against *Xoo* in rice. If vertical resistance genes are segregating in the experimental population, these genes could mask the effect of horizontal genes. To our knowledge, there have been no reports of genetic analysis of *Xoo* resistance targeting only horizontal resistance using two susceptible cultivars. For this study, the difference in the reaction to *Xoo* between KO and AR was analyzed using QTL mapping.

## 2. Results

### 2.1. Phenotypic Evaluation of Parental Lines and F_2_ Population to Progression of Lesion Length to Xoo

We confirmed the reaction of KO and AR to five Japanese *Xoo* races (Table 1): KO displayed shorter lesion length after inoculation of all the *Xoo* races tested and eventually stopped the lesion progression after 10–18 cm, whereas AR displayed longer lesion length, exceeding 40–54 cm.

**Figure 1:**
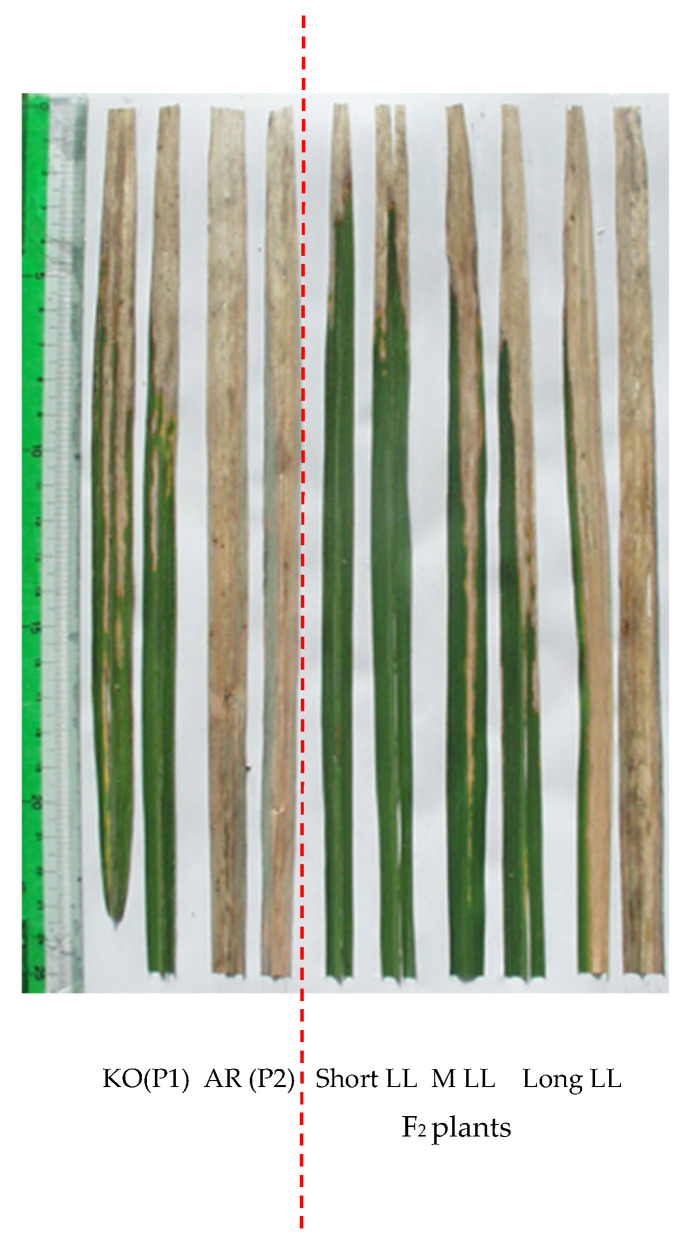
Morphology of lesion length at 18 days for KO (parent 1 (P1)) and AR (parent 2 (P2)) and its F_2_ progenies inoculated to Race II *Xoo* (strain T7147) showing short lesion length, moderate lesion length (MLL), and long lesion length.

**Figure 2:**
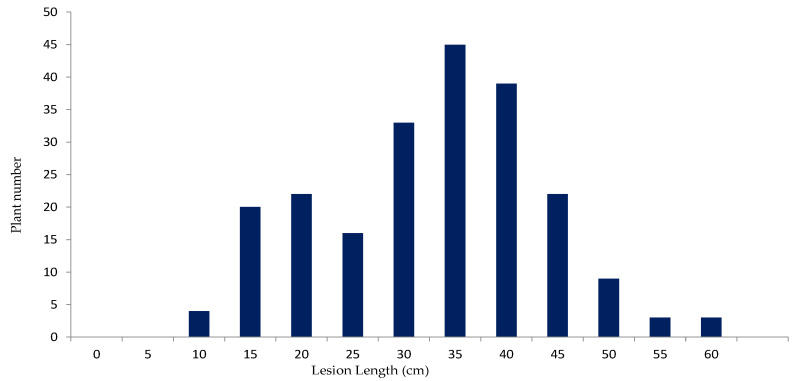
Frequency distribution of lesion length after inoculating with *Xoo* Race II (strain T7147) using leaf-clipping method after 18 days in the F_2_ population (*n* =216) derived from the cross between KO and AR. Three leaf measurements from each plant were recorded. An average of three leaves was used as a phenotypic characteristic.

To dissect the quantitative difference in the reaction to *Xoo* genetically, the population from the cross between KO and AR was subjected to inoculation to Race II strain (Figure 1), which was frequently used in our earlier studies [19,30,31] as a tester *Xoo* in genetic analysis of *Xoo* resistance in rice. The F_2_ population derived from the cross between the two cultivars represented continuous distribution of 5.5–60 cm (Figure 2).

### 2.2. Putative Quantitative Trait Loci (QTL) Mapping for Resistance to Bacterial Blight (BB)

**Figure 3:**
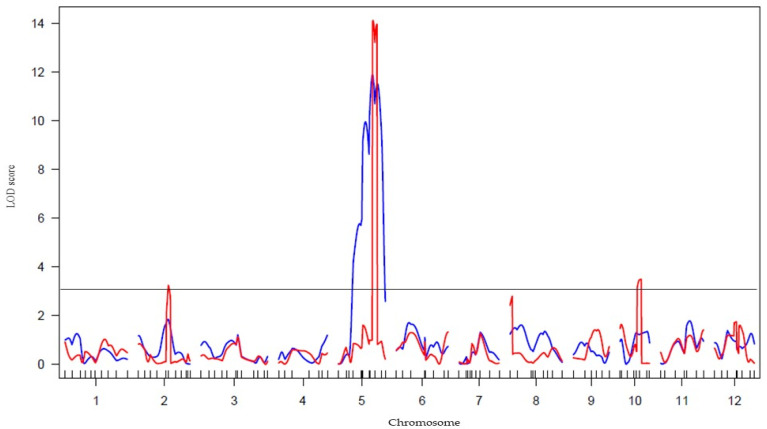
Plotting of LOD scores for QTL analysis at 99% tile threshold using “hk” regression method. The blue line data were calculated using simple interval mapping (SIM). The red line data show calculated LOD using a composite interval mapping (CIM) approach with stepwise covariate addition within the regression model.

**Figure 4:**
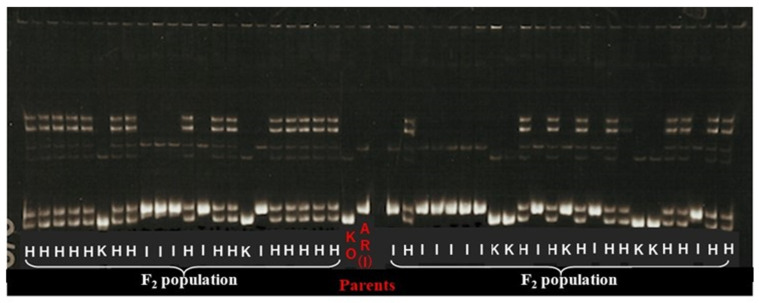
Banding pattern of linked marker E60663 on chromosome 5 with the highest likelihood ratio of putative QTL under SIM and CIM. K represents Koshihikari banding profile; H is denoted as heterozygous while I banding pattern follows ARC7013 in F_2_ population. In the middle of the gel, a control ladder containing both parents is loaded and labelled as red.

**Figure 5:**
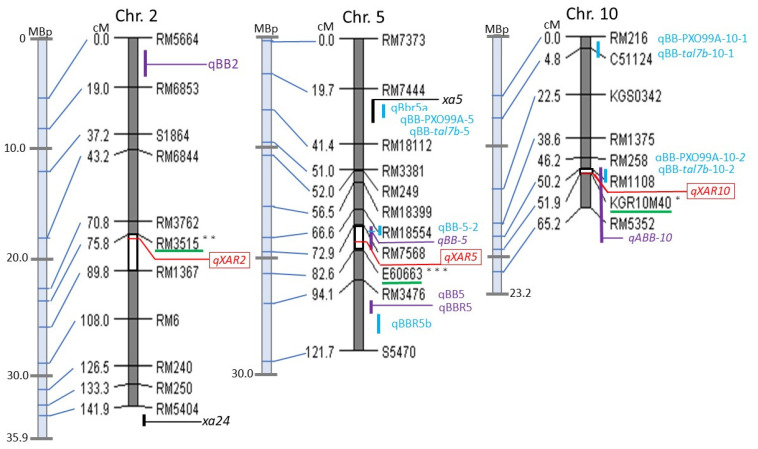
An associative map comparing the QTLs against *Xoo* pathogen in this study with *Xoo* resistance genes published before. The left vertical categorized light blue thick lines represent the Nipponbare physical map [33] of chromosomes 2, 5, and 10 and are linked to the genetic map on the right showing DNA markers for QTLs controlling elongation of lesion length, which is constructed using AntMap [34] with the Kosambi function [35]. The chromosomes are represented as dark grey bars with SSR, InDel markers used in this work are presented on the right side. The genetic distance (cM) is indicated at the left of the bar. QTLs found in this study are shown as red boxes; the Bayesian 99% interval of QTLs is represented as filled-in white within the chromosome bars. The nearest genetic marker to QTLs discovered in this study is underlined with green. Markers that are significant at the 0.001, 0.01, and 0.05 probability level are marked with ***, **, *, respectively. The previously published QTL genes that are not fine mapped are labelled as vertical blue and purple lines indicating the confidence interval. *R* resistance genes associated with BB resistance are shown as black lines on chromosomes of interest for this study.

Out of more than 2000 genetic markers in our lab database, 113 DNA markers were tested, including SSR, STS, and InDel PCR-based markers which were pre-surveyed and screened for polymorphism first between *japonica* and *indica* cultivars that were evenly distributed on 12 chromosomes. From these, 80 markers that provided good results were selected while 27 markers were reselected from our stock because distinguishing between genotypes was difficult. This survey allowed us to select the best 107 DNA markers to carry out genetic analysis for KO and AR. Among them, 107 genetic markers covering all 12 chromosomes produced clear, reproducible polymorphic and co-dominant banding patterns and were selected for further study (Supplementary Materials: Table S1). Then, a linkage map covering all 12 chromosomes was constructed (Supplementary Materials: Figure S1). This linkage map was used for QTL analysis. QTL analysis of lesion length elongation with the obtained linkage of 107 DNA markers (Table S1) was applied to the F_2_ population described above using R/qtl by adopting simple interval mapping (SIM) based on maximum likelihood and composite interval mapping (CIM) approaches. Three QTLs controlling lesion length were detected on chromosomes 2, 5, and 10 (Figure 3) using both SIM and CIM, with a potential candidate on chromosome 8. Figure 4 illustrates the banding pattern displayed by the highest LOD score on chromosome 5 highlighted in Figure 3 from segregating the population on the flanked nearest genetic marker of E60663. These three QTLs were designated respectively as *qXAR2* (QTL for *Xoo* resistance on chromosome 2), *qXAR5*, and *qXAR10* (Figure 5) [32]. The first, *qXAR2*, was located between marker RM3515 and RM1367, flanked to the nearest marker RM3515. This QTL explained 3.9% of the phenotypic variance and had the logarithm of odds (LOD) score of 2.4 derived from the SIM method. Under the CIM approach, the LOD score was 3.1, located at the map region of 75.8 cM on chromosome 2. In fact, *qXAR5* had the highest LOD score of 14.0 defined by the CIM approach and 11.6 from the SIM method, explaining approximately 20.5% of the total resistance variance. It was mapped close to 79.0 cM between markers RM7568 and E60663 on chromosome 5 flanked by the nearest marker of E60663. *qXAR10* was mapped to a region of 55.0 cM flanked by markers RM1108 and KGR10M40 on chromosome 10 and the nearest marker was KGR10M40. The LOD scores of *qXAR10* under the CIM and SIM approaches were 3.4 and 1.4, respectively, accounting for 2.3% of the phenotypic variance (Table 2).

A comparative genetic map was constructed by calculating the 99% confidence interval of putative QTLs (Figure 5). The largest confidence level was on *qXAR2*, which was approximately 14 cM, located in the region of RM3515–RM1367. Among the three identified QTLs, *qXAR5* and *qXAR10* had the short confidence intervals. *qXAR5* and *qXAR10* had an approximately 10 cM interval region in markers RM7568–E60663 and 1.7 cM in RM1108–KGR10M40.

### 2.3. Alleles Contributing to Short Lesion Length in the Targeted Regions

The expression level of the allelic effect at the nearest marker, RM3515, on chromosome 2 is −2.8, which results in the KO genotype being skewed towards shorter lesion length, whereas AR is distributed along the longer lesion length. This finding indicates that the KO homozygous *qXAR2* might reduce the lesion length by 5.6 cm compared to AR homozygotes. 

**Figure 6:**
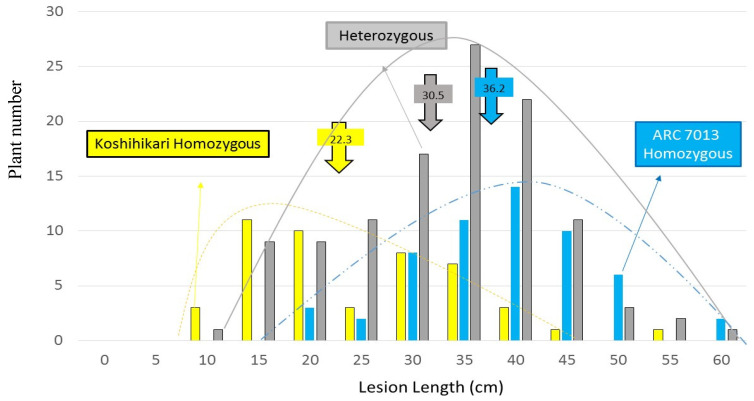
Frequency distribution of lesion length genotypic expression on chromosome 5 after PCR analysis of F_2_ progenies derived from KO × AR, which gave the maximum strength of evidence for the presence of QTL at this position using both SIM and CIM methods. The classified genotypes were assessed using E60663 as indicated: yellow, homozygous for KO; grey, heterozygous; blue, homozygous for AR.

From chromosome 5, given the highest LOD score flanked by marker E60663 using both the SIM and CIM method at statistical significance level of *p* ≤ 0.01, we determined that the KO allele contributed towards shorter lesion length in the F_2_ population. The additive effect of *qXAR5* is −7.0, indicating that KO homozygous might contribute to reducing the lesion length by 14.0 cm. The genotypic distribution on flanked marker E60663 on chromosome 5 also provided evidence that KO’s genotype was skewed clearly towards shorter lesion length when compared to AR, which was skewed more towards longer lesion length (Figure 6).

It is particularly interesting that compared to chromosomes 2 and 5, chromosome 10 had the opposite genotypic distribution: AR skewed towards the shorter lesion length; KO skewed towards the longer lesion length. The additive effect *qXAR10* is 2.3, thereby indicating that the AR allele homozygote might reduce the lesion length by 4.6 cm, as shown in Table 2. Moreover, the dominance action and its interactions on the same locus at *qXAR2* and *qXAR5* indicate that the heterozygote genotypic value resembles that of AR homozygotes, whereas *qXAR10* showed a smaller dominance effect. 

## 3. Discussion

Bacterial leaf blight (BB) caused by the *Xoo* pathogen is regarded as a devastating and widespread disease affecting rice in parts of the world where rice is mostly cultivated. The approximately 42 resistance genes to BB which have been identified are increasingly being used in breeding programs [31,36,37]. Because these resistant genes have been used in elite cultivars for decades, they make the cultivars highly vulnerable to evolving *Xoo* pathotypes, thereby necessitating the reservoir of rice germplasm against them.

For this study, QTL analysis was performed using the F_2_ population derived from the cross between an elite *japonica* cultivar, KO [38], and an *indica* cultivar, AR. Neither has true resistance genes. Both are susceptible to BB disease, making this study and its findings even more vital. Moreover, previous research is mostly based on using one resistant and one susceptible material which does not provide a good reference point of true horizontal QTLs in most instances. F_2_ generation was initially used in our study, in order to first determine and ascertain statistical difference in early generation between two susceptible cultivars. As two susceptible cultivars were involved, it was important to discover that the observed phenotypic characteristics were actually due to polygenic gene interaction. This will also form the basis as a screen process to further develop our recombinant inbred line and chromosomal segment substitution line materials. This method will also serve as an early screening induction signal, when using purely susceptible cultivars, to better isolate planting material lines for future research. KO phenotypic characteristics when exposed to *Xoo* indicate that it has the potential to reduce the progression of the foliar lesion, which provides an understanding of the resistance against *Xoo*. The F_2_ population showed evidence of short lesion length against Japanese Race II (strain T7147) isolate. After analyzing the genotypic data and phenotypic characteristics of the F_2_ population, it was discovered that lesion elongation against BB disease was controlled by QTLs. Using composite interval mapping (CIM), *qXAR2* on chromosome 2, *qXAR5* on chromosome 5, and *qXAR10* on chromosome 10 were detected, although only one *qXAR5* reached the 99% significance threshold during simple interval mapping (SIM). In a CIM model, results calculated using the SIM fitqtl function [39] contributed further to significant phenotypic variance (2.3–20.5%) with 26.7% of the total resistance variance. Earlier reports have also described QTLs that have been discovered and mapped to chromosomes 1, 3, 4, 5, 7, 8, 9, 10, 11, and 12 [40,41], to chromosomes 1, 3, and 5 [8], to chromosomes 2, 3, 5, 7, and 8 [27], and to all chromosomes [28]. Among them, two studies, [8] and [40], used PCR-based DNA markers for which primer sequences were available, thereby enabling comparative analysis of this study and the two earlier studies [8,40].

As reported earlier, *xa5* has been mapped at an interval of 0.5 cM between markers RS7 (411 kb region on chromosome 5 of Os-Nipponbare-Reference-IRGSP-1.0 [42]) and RM611 (488 kb region) [43]. Actually, the *qXAR5* identified in this study is also mapped to chromosome 5, but its mapping region differed. *qXAR5* was mapped physically between markers in RM7568 (19,490 kb; 72.9 cM) and E60663 (21,204 kb; 82.6 cM) regions, which provided the highest LOD score at the position interval of 79.0 cM in this study. Han et al. located one *Xoo* resistance QTL named qBBR5 between RM7081 (24,587 kb) and RM3616 (26,344 kb) on chromosome 5 [8]. This finding indicates that both *qXAR5* and qBBR5 are located at different positions on chromosome 5. Additionally, the genetic background used in both studies is also a factor contributing to this difference. The allele from a wild rice species *O. meyeriana* has a strong effect on *Xoo* resistance among the three detected QTLs.

Djedatin et al. detected one QTL, named *qBB-5*, which was closely linked with a DNA marker RM440 (19,975 kb) [40], located between RM7568 and E60663. The 79.0 cM highest LOD score of *qXAR5* is located near E60663. The tropical *japonica* cultivar Azucena carries a resistant allele against PXO86, a *Xoo* race from the Philippines. In addition, an *indica* cultivar IR64 carries a susceptible allele. Although their exact chromosomal locations have remained unknown, the results of rough mapping suggest that *qXAR5*, qBBR5, and *qBB-5* mutually differ.

*qXAR2* is located between RM3515 (24,022 kb) and RM1367 (27,065 kb). After Wu et al. conducted fine mapping of an *Xoo* resistance gene *xa24*, they reported that the *xa24* gene is located between two DNA markers on the distal end of the long arm of chromosome 2: RM14220 (35,679 kb) and RM14226 (35,781 kb) [44]. The physical locations differ from one another: they are not allelic. No other report describes *Xoo* resistance genes or QTLs mapped on chromosome 2. If genetically dissected into a single gene, *qXAR2* will be a new *Xoo* resistance gene.

*qXAR10* is located between RM1108 (19,233 kb) and KGR10M40 (19,469 kb) on chromosome 10. No Mendelian *Xoo* resistance gene has been reported on chromosome 10. Djedatin et al. detected one QTL named *qABB-10* as closely linked with a DNA marker RM294A [40]. Actually, the primer sequence information of RM294A indicates that it is located on chromosome 1 of the latest rice genome sequence [45]. RM294A is flanked by two markers: RM171 and RM228 [40]. RM171 and RM228 are located respectively at 19,120 kb and 22,314 kb on chromosome 10. The approximate locations of *qXAR10* and *qABB-10* are overlapping. However, their gene actions differ: results of the present study show that a *japonica* cultivar KO carries a susceptible allele against Japanese *Xoo* race II and that *indica* cultivar AR carries a resistant allele on *qXAR10*. In the case of *qABB-10*, the tropical *japonica* cultivar Azucena carries a resistant allele against NAI8 [40], a *Xoo* race from Niger, and an *indica* cultivar, IR64, carries a susceptible allele on *qABB-10* [40]. These results suggest that *qXAR10* and *qABB-10* differ, although precise mapping of both genes will be necessary to reach a conclusion.

According to a review by Vikal et al., most *Xoo* resistance genes identified to date show hyper-sensitive response to pathogens and inhibit their intrusion by programmed cell death [36]. In fact, KO shows no hyper-sensitive response. Therefore, it is likely that the QTLs detected in this study exert resistance using different mechanisms. Recently, *Xa4*, a durable *Xoo* resistance gene, and *pi21*, a field resistance gene to rice blast, reportedly have atypical resistance mechanisms: Hu et al. reported that *Xa4* encodes a cell-wall associated kinase, enhancing resistance to bacterial infection by strengthening the cell wall via promotion of cellulose synthesis and suppression of cell wall loosening [46]. Another atypical resistance mechanism is reported in *pi21*: the wild type (susceptible) allele *Pi21* encodes a proline-rich protein that includes a putative heavy metal-binding domain and putative protein–protein interaction motifs [47]. This allele appears to slow the plant’s defense responses, which might support optimization of defense mechanisms. Deletion in its proline-rich motif in the resistant allele *pi21* inhibits this slowing. Fonseca and Mysore [48] listed genes involved in nonhost disease resistance. These genes also might contribute to horizontal resistance.

Previous research is mostly based on using one resistant and one susceptible material which do not provide a good reference point of true horizontal QTLs in most instances. This study highlighted the existence of *Xoo* resistance genes without hyper-sensitive responses, which are mostly due to the QTL mapping population of which parental cultivars carry no major vertical resistance genes against *Xoo*. Therefore, we could detect three QTLs with 107 DNA markers, which is a relatively small number compared to what can be detected with next generation sequencer-based methods. To our knowledge, there have been no previous reports highlighting this aspect of study and this may be the first report of genetic analysis of *Xoo* resistance targeting only horizontal resistance. Furthermore, 107 markers could be enough for a starting point of further linkage analysis using near-isogenic lines as described below.

The three QTLs examined for this study might contribute to new mechanisms of resistance against *Xoo*. Among the three QTLs identified, the KO’s allele of *qXAR5* confers the strongest resistance against *Xoo*. Therefore, we are undertaking further study of *qXAR5*. The development of near-isogenic lines of *qXAR5* under the IR24 genetic background, of which many *Xoo* resistance genes have been introduced and induced by mutation [49,50], is undertaken to perform fine mapping of *qXAR5* for comparison and to combine *qXAR5* with other genes to assess their resistance against multiple *Xoo* races under the same genetic background. The present study revealed that even small or minor QTLs, such as *qXAR2* and *qXAR10*, might eventually be able to defend against the *Xoo* pathogen. The minor QTLs can also be used in pyramiding resistance with *R* genes to breed more durable and resistant cultivars. This pyramiding has also been confirmed in an earlier study done by Hu et al., which described that although minor QTL genes are less efficient at controlling diseases than *R* genes are, pyramiding two or more minor QTL genes has some potential to help in breeding rice cultivars that are resistant to BB diseases [26]. Therefore, precise mapping of *qXAR2* and *qXAR10* is also undertaken.

## 4. Materials and Methods

### 4.1. Plant Materials and Bacterial Race Preparation

Koshihikari (KO) is a popular Japanese *japonica* rice cultivar. An *indica* rice cultivar from India is ARC 7013 (AR). Their reactions to *Xoo* were tested in 2008 (Table 1). Plants were inoculated in the manner described below for each cultivar. Then, a cross between KO and AR was conducted to create F_1_ plants. In 2008, an F_2_ population was produced for genetic analysis and for ascertaining the location of putative QTLs. The F_2_ segregating population from germinated seeds crossed between KO and AR was seeded in a seedling box containing sterilized soil left in a glasshouse for two weeks, half submerged in clean water. During the third week, 300 healthy seedlings were transferred outside of the glasshouse. About one month after the sowing date, they were transplanted by hand to a paddy field at the Kagoshima University Experimental Field. The field was filled with fresh clean water. Planting was done with spacing of 15 cm within rows and 30 cm between rows.

According to virulence and origin, *Xoo* are differentiated into many races. Five Japanese races were used for this study, designated as race I (strain T7174), race II (strain T7147), race III (strain T7133), race IV (strain H75373), and race V (strain H75304). The isolate race II was used for QTL analysis. The *Xoo* isolates were kept under −20 °C in skim milk medium (SMM). The SMM, which included 10% skim milk and 1.5% sodium glutamate, was sterilized at 121 °C for 20 min. A similar method, with minor modification to that reported by Wakimoto [51], was used to prepare potato semi-synthetic agar medium (PSA) media to culture the bacteria. Before field inoculation, a smear loop (5 mm diameter) was used to transfer two scoops of bacteria inoculum from the stock culture tube to the PSA media. The bacteria were cultured for 48 h at 28 °C. Two hours before inoculation was conducted in the rice field, the bacterial cells were suspended in distilled water. The suspension’s absorbance frequency was calibrated using the spectrophotometer: *A* = 0.05 or 620 nm, which had approximate concentration of 10^8^ cfu/mL. Mapping population plants were inoculated at the booting stage with the suspension using leaf clipping method [52,53]. At 18 days after inoculation, each plant’s reaction to the *Xoo* pathogen was evaluated by lesion length: lesions (cm) of three leaves from each plant were measured using a ruler; their mean value was adopted as phenotypic data.

### 4.2. Genomic DNA Extraction and PCR Analysis Using DNA Markers

One week before inoculation, leaf samples from each F_2_ plant were collected for genomic DNA extraction. DNA was extracted using the method described by Dellaporta et al. with a few modifications [54]: each leaf tip of 2 cm long was collected in 2.0 mL micro centrifuge tube from a single plant and was stored at −20 °C. Later, each leaf was vacuum dried before DNA extraction began. Then in each tube, two 5-mm-diameter stainless steel balls were added in order to break the leaf cellulose tissues and grind the leave into powdery form to maximize DNA quantity. The tubes were then transferred to aluminum block with 2 mL deep well. They were covered with a hard lid and shaken hard in ShakeMaster (BioMedical Science Inc., Tokyo, Japan) for 2 min to completely grind the leaf samples. Then, 500 μL extraction buffer (1% SDS, 100 mM Tris-HCl (pH 8.0), 50 mM EDTA (pH 8.0), 500 mM NaCl) was added to the samples and thoroughly mixed. After mixing, the samples were incubated for 60 min at 60 °C. Next, 150 μL of 5 M potassium acetate was added after incubation period and mixed thoroughly by hand, then the samples were placed on ice for 30 min. Each sample tube was vortexed for approximately 5 s to remove any trapped air bubbles which may have been floating within the samples. Samples were centrifuged for 15 min at 4 °C at 4200 rpm (3600× *g*) using swing rotor centrifuge. Then, 300 μL supernatant was pipetted into the new 1.5 μL Eppendorf tube and the same amount of isopropanol was added to the samples and gently mixed. After which, samples were centrifuged for 10 min at room temperature. Then the supernatant was discarded, and tubes were placed invertedly on clean paper towel to dry. DNA pellet was rinsed with 500 μL of 70% ethanol and left to settle for 10 min, followed by centrifuge for another 5 min at room temperature at 4200 rpm. Supernatant was gently discarded and DNA pallets were dried. Later, 100 μL of TE–RNase were added into each tube of DNA pallets and dissolved by incubating the samples for 5 min at 60 °C. The DNA concentration was quantified using a spectrophotometer (Nanodrop 8000; Thermo Fisher Scientific Inc. Wilmington, DE, USA). For this study, 107 simple sequence repeat (SSR) and insertion/deletion (InDel) markers were acquired from earlier publications [31,55,56,57,58,59,60,61,62] (Supplementary Materials: Table S1). The polymerase chain reaction (PCR) cocktail mixture (4.5 µL) comprised genomic DNA 10 ng, dNTPs 200 µM, 10 × buffer containing MgCl_2_, individual DNA markers 0.2 µM, distilled H_2_O 1.6 µL, and Taq polymerase 0.25 U. The amplified PCR product was separated using gel electrophoresis (PAGE) on 10% polyacrylamide gels in 1 × TBE solution for 1 h followed by staining gels with ethidium bromide for 10 min and later gels were washed with distilled water for another 30 min. Genotype visualization photo recording was performed under UV light (GelDoc–It^®®^ TS Imaging system; UVP LLC, Upland, CA, USA). DNA profiling of 216 segregating F_2_ populations derived from KO and AR was individually evaluated based on the band display pattern of the mapping population. Each line was scored by having the linkage marker pattern that corresponded to one of the parents, as shown in Figure 4. Moreover, the genotyping matrix was set up according to the displayed bands which was recorded as: Koshihikari homozygous–K, Heterozygous patterns from both parents–H, and ARC7013 homozygous–I (representing *indica*). K, H, and I were later matrixed to A, H, and B, respectively, during R/qtl coding stage. 

### 4.3. Mapping Putative QTLs against Resistance to BB Disease

For this study, QTL analyses were conducted using R software programming language ver. 4.0.3 (https://rqtl.org/download/ (accessed on 12 June 2021)) with an add-on qtl library package [63] downloaded from comprehensive R archive network (https://cran.r-project.org/ (accessed on 12 June 2021)). An F_2_ intercross was conducted using R/qtl with 216 F_2_ individuals and 107 markers, declaring missing genotype data as (na.strings= “-”). In addition, for this study, to carry out precise location of the QTLs, simple interval mapping (SIM) and composite interval mapping (CIM) using stepwise scanning at 1 cM were used to discover QTLs via “hk” regression method and error probability assigned as 0.01. Then CIM was used to eliminate the probability of being able to detect “ghost QTLs” on a wide genome scan by Bayes 99% threshold via permutation test.

Before moving further, genotype data were assessed for problematic markers by inspecting the F_2_ segregation ratio having a 1:2:1 likely pattern. Main effects of QTL were estimated using the function logarithm of odds (LOD) having maximum likelihood estimation of all 12 autosomes. A permutation test set to 1000 iterations was applied to estimate the genome-wide significance level of LOD threshold equal to *p* ≤ 0.01 for the trait/chromosome combination (log-likelihood of odds (LOD) score of more than or equal to 3). R/qtl was used to calculate the 99% confidence estimate interval that had significant QTLs at highlighted chromosomes using Bayes credible interval approach, *f* (*θ* | data) = 10^LOD(*θ*)^/Σ*_θ_*^10LOD(*θ*)^. A genetic map was developed using AntMap software. The linkage mapping distances were calculated using recombination frequency by utilizing Kosambi mapping function with 5000 iterations, with *r* = 0.005 [25,26]. 

## 5. Conclusions

*Xanthomonas oryzae* pv. *oryzae* (*Xoo*), an economically important pathogen, causes bacterial leaf blight disease in rice, an important cereal crop that supports and feeds about half of the Earth’s population. Global climatic conditions will continue to worsen in the coming years because of global warming. Continued increases in weather temperatures may provide favorable conditions for the *Xoo* pathogen to evolve into more virulent strains. Resistance genes might lead to future breakdown, leaving a higher incidence of elite rice cultivars becoming more prone to BB disease. Therefore, searching for new and durable resistant genes that are useful for breeding programs to develop elite cultivars with desirable agronomic traits is of paramount importance. From the cross between *japonica* KO and *indica* AR, findings from this study show that the F_2_ population exhibits continuous distribution to the *Xoo* (strain T7147). Three QTLs were discovered on chromosomes 2, 5, and 10, with LOD scores of 3.1, 14.0, and 3.4 using the CIM method and 2.4, 11.6, and 1.4 using the SIM method, respectively. The highest LOD is on chromosome 5, flanked by the nearest marker E60663 at position 79.0 cM. Additionally, chromosomes 2 and 5 show that the KO allele contributes to shorter lesion length. However, chromosome 10 provides an interesting differential of having AR as the shorter lesion length. This finding helps to elucidate the phenomenon of KO’s shorter lesion length than that of AR. The QTLs identified in this study can facilitate better understanding of the genetic and morphological fundamentals necessary to develop horizontal resistant rice varieties that can withstand evolving pathogens in the field. Further studies will be conducted with multiple strains of *Xoo* pathogens to extend this research and to test the field resistance in the KO cultivar.

## Figures and Tables

**Table 1:** Average of lesion length (cm) reactions of rice accessions: Koshihikari and ARC7013 after inoculation with five Japanese races of *Xanthomonas oryzae* pv. *oryzae.*

	Lesion Length (cm)				
Rice Accession	Race I T7174	Race II T7147	Race III T7133	Race IV H75373	Race V H75304
Koshihikari Japan	14.9	13.5	13.5	10.1	17.7
ARC7013 India	40.5	48.5	43.3	43.3	54.0

**Table 2:** QTLs controlling lesion length after inoculation of *Xoo* race II.

QTL	Chr.	Nearest Marker	Lod Scores Using (SIM) ^a^ & (CIM) ^b^	QTL cM (CI) ^c^	*R*^2^% SIM ^d^	Additive Effect ^e^	Dominance Effect ^f^	DPE ^g^
*qXAR2*	2	RM3515	(2.4) ^a^; (3.1) ^b^	75.8 (75.8–89.8)	3.9	−2.8	2.5	KO **
*qXAR5*	5	E60663	(11.6) ^a^; (14.0) ^b^	79.0 (72.9–82.9)	20.5	−7.0	1.8	KO ***
*qXAR10*	10	KGR10M40	(1.4) ^a^; (3.4) ^b^	51.0 (50.2–51.9)	2.3	2.3	0.4	AR *

^a^ Simple interval mapping for LOD score of putative QTLs; ^b^ Composite interval mapping for detecting multiple QTLs on a genome; ^c^ CI, Confidence interval, 99% confidence interval for detected QTLs location on the chromosome region; ^d^ The proportion of variance explained by the QTL; ^e^ Negative and positive standards indicate additive lesion length-inhibiting effect contributed by KO and AR alleles, respectively; ^f^ Positive values mean dominance toward AR alleles; ^g^ Direction of phenotypic effect where KO and AR indicate that the Koshihikari and ARC7013 alleles reduce lesion progression values, respectively. Markers that are significant at the 0.001, 0.01, and 0.05 probability level are denoted as ‘***’, ‘**’, and ‘*’, respectively.

## Data Availability

The dataset collected from this research and all genetical analyzed work together with the statistical software coding are available from the corresponding author upon reasonable request.

## Supplementary Materials

The following are available online at https://www.mdpi.com/article/10.3390/plants11070867/s1, Figure S1: An associative map comparing the QTLs against *Xoo* pathogen in this study. Table S1: DNA markers used for linkage analysis of F_2_ plants from the between Koshihikari and ARC7013.
