# Supplementary material for: QTL Analysis Revealed One Major Genetic Factor Inhibiting Lesion Elongation by Bacterial Blight (Xanthomonas oryzae pv. oryzae) from a japonica Cultivar Koshihikari in Rice
Source: Plants (Basel). 2022 Mar 24;11(7):867. doi: 10.3390/plants11070867 (PMC9003242; doi:10.3390/plants11070867)
Supplement: Supplementary file 1 [file plants-11-00867-s001.zip › Figure S1. Genetic map_Physical map_All Chrs.pdf]

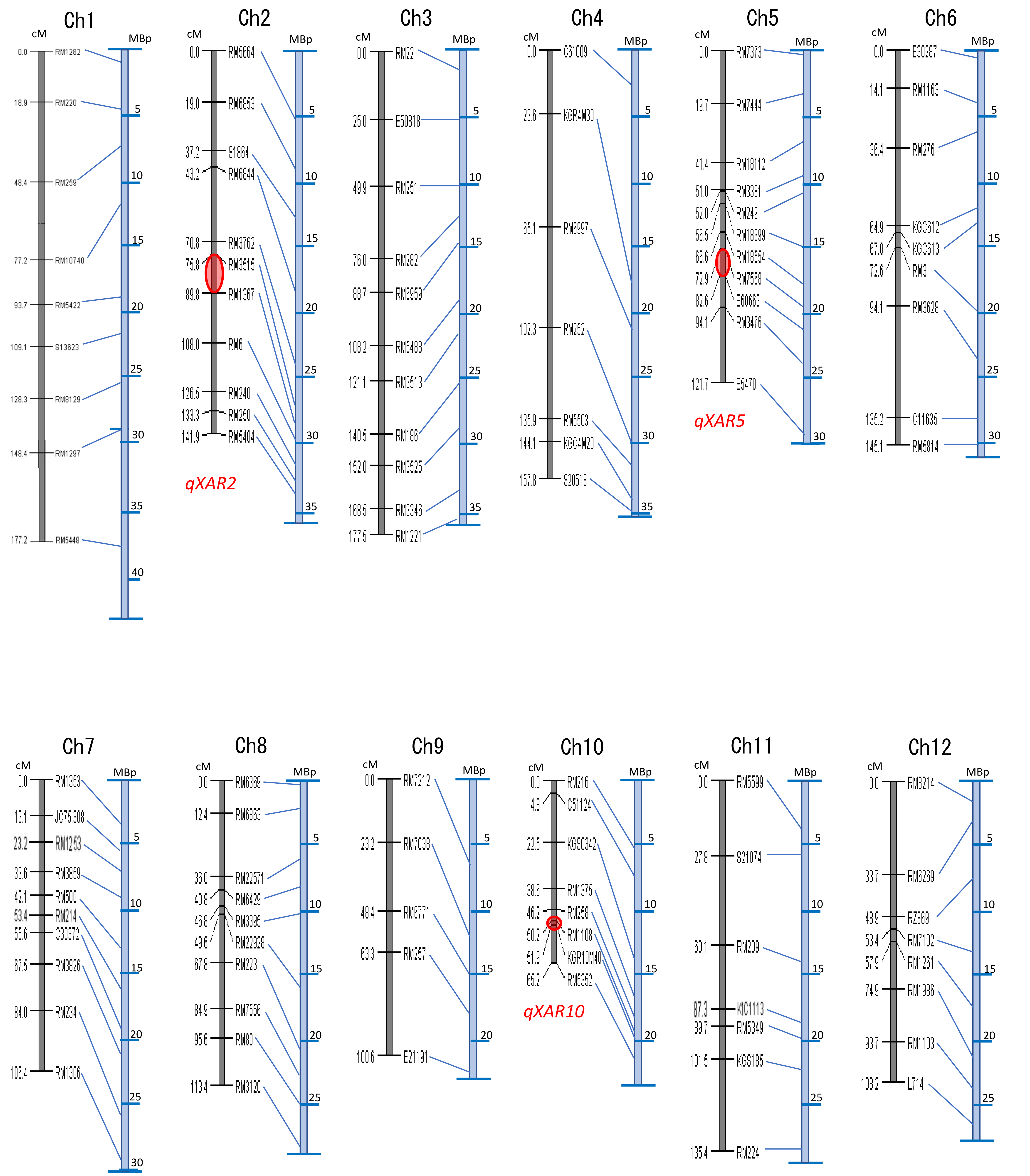

Figure S1. An associative map comparing the QTLs against *Xoo* pathogen in this study. The right vertical categorized light blue thick lines represent the Nipponbare physical map [25] of all 12 rice chromosomes and is linked to the genetic map on the left showing DNA markers for QTLs controlling elongation of lesion length is constructed using AntMap. The chromosome represented as dark grey bars with SSR; InDel markers used in this work are presented on the right side of the bar. The genetic distance (cM) is indicated at the left of the bar. QTLs found in this study are shown as circled red.
